# NLRC5‐Deficient Macrophages Promote a Tumor‐Permissive Phenotype via AXL‐ and MERTK‐Mediated Efferocytosis

**DOI:** 10.1096/fj.202504879R

**Published:** 2026-08-01

**Authors:** Shambel Araya Haile, Alessandra Gatta, Nina Colon, Dana J. Philpott, Thomas A. Kufer, Le Ying, Dongmei Tong, Richard L. Ferrero

**Affiliations:** ^1^ Centre for Innate Immunity and Infectious Diseases Hudson Institute of Medical Research Clayton Victoria Australia; ^2^ Department of Molecular and Translational Science Monash University Melbourne Victoria Australia; ^3^ Department of Veterinary Sciences Università di Pisa Pisa Italy; ^4^ Department of Immunology University of Toronto Toronto Ontario Canada; ^5^ Department of Immunology, Institute of Nutritional Medicine University of Hohenheim Stuttgart Germany; ^6^ Department of Microbiology, Biomedicine Discovery Institute Monash University Melbourne Victoria Australia

**Keywords:** efferocytosis, gastric B‐cell lymphoma, *Helicobacter pylori*, NLRC5, tumor‐associated macrophages

## Abstract

The innate immune protein NLRC5 plays a key role in cancer immune surveillance. Reduced *NLRC5* expression is associated with a poor prognosis for many types of cancers. Previously, we showed that mice with a myeloid‐specific deletion of *Nlrc5* (*Nlrc5* ^mø‐KO^) develop gastric lymphoid lesions to *Helicobacter* infection resembling early‐stage marginal zone lymphoma. We hypothesized that NLRC5 deficiency may promote a tumor‐permissive microenvironment mediated by tumor‐associated macrophages (TAMs). Consistent with this hypothesis, splenic macrophages from *Helicobacter*‐infected *Nlrc5* ^mø‐KO^ mice had upregulated expression of genes encoding the TAM receptor tyrosine kinases, Axl and Mertk. The levels of *AXL* and *MERTK* gene expression and MERTK phosphorylation were increased in *NLRC5* ^−/−^ THP‐1 macrophages when compared with WT cells. In response to *Helicobacter* stimulation, *Nlrc5* ^*−/−*^ macrophages had significantly elevated anti‐inflammatory responses (IL‐10, TGF‐β, *Socs1*, *Socs3*) compared with WT cells. Importantly, *Nlrc5* ^*−/−*^macrophages showed enhanced efferocytosis and reduced antigen presentation to CD8^+^ T cells. Pretreatment of macrophages with AXL and MERTK inhibitors (R428, UNC2025) resulted in reduced efferocytosis and phosphorylation of downstream signaling molecules, STAT3 and ERK1/2. We propose that defective NLRC5 signaling in macrophages leads to tumor‐permissive responses, thereby promoting the development of gastric lymphoid neogenesis to *Helicobacter* infection.

## Graphical abstract

Defective NLRC5 signaling in macrophages drives TAM phenotype and anti‐inflammatory responses via STAT3/ERK, leading to tumor‐permissive conditions and gastric lymphoid neogenesis during *Helicobacter* infection.

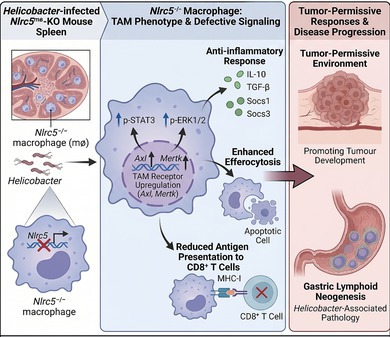

## 1 Introduction

*Helicobacter pylori* is a bacterial class‐I carcinogen [1, 2] that colonizes the human gastric epithelium and is the leading cause of infection‐related cancer [1, 3]. Chronic *H. pylori* infection is implicated in over 90% of cases of gastric mucosa‐associated lymphoid tissue (MALT) lymphoma, the most common type of extra‐nodal marginal zone B‐cell lymphoma [4]. Although the normal gastric mucosa lacks organized lymphoid tissue [5, 6], persistent *H. pylori* infection induces chronic inflammation that can lead to the formation of ectopic MALT in a subset of individuals, ultimately progressing to low‐grade gastric B‐cell lymphoma [4, 6]. Sustained antigenic stimulation from *H. pylori* drives T cell‐dependent clonal B‐cell expansion and gene translocations, contributing to lymphomagenesis [4, 7]. Although the genetic alterations involved are well characterized, the host immune mechanisms driving MALT type B‐cell lymphoma remain less well understood.

Murine models of *Helicobacter*‐induced MALT lymphoma reproduce the human disease; however, lymphomagenesis occurs slowly (12–24 months) [8, 9]. We developed a mouse model in which animals develop MALT lesions at 3 months postinfection, providing a more practicable system to study gastric MALT lymphomagenesis [10, 11, 12]. These mice have a myeloid‐specific deletion of the innate immune protein Nod like receptor (NLR) family CARD domain containing 5 (NLRC5), which has been reported to play an important role in antitumor immunity through its regulation of classical major histocompatibility complex class‐I (MHC‐I) antigen presentation and CD8^+^ T cell responses [13, 14, 15]. Conditional *Nlrc5* knockout mice (*Nlrc5* ^mø‐KO^) exhibit characteristics of the human disease, including B‐cell follicles invading the gastric epithelium to form lymphoepithelial‐like lesions and the presence of centrocyte‐like cells [11]. Additionally, gastric tissues from the *Nlrc5* ^mø‐KO^ mice also had features of an immunosuppressive microenvironment, characterized by forkhead box P3 (FoxP3)^+^ T‐regulatory cell infiltration and expression of programmed cell death protein 1 (PD‐1) and programmed death‐ligand 1 (PD‐L1) [11], markers associated with tumor‐associated macrophages (TAMs) [16]. Interestingly, it was previously proposed that TAMs, a subset of immunosuppressive type 2 myeloid cells with tumor‐promoting functions [17], may play a role in *Helicobacter*‐dependent gastric MALT lymphomagenesis [18].

In this study, we demonstrated that splenic macrophages from *Nlrc5* ^mø‐KO^ mice exhibit the upregulated expression of genes encoding Axl receptor tyrosine kinase (Axl) and myeloid epithelial reproductive tyrosine kinase (Mertk), key receptor tyrosine kinases expressed by TAMs. TAMs migrate from the spleen to tumor sites in response to inflammation [19] where they mediate immunosuppression via binding to their ligands, growth arrest specific protein 6 (GAS6) and Protein S (PROS1) [17, 20]. These interactions were reported to suppress toll‐like receptor (TLR)‐induced cytokine production through the type I interferon receptor (IFNAR)/signal transducer and activator of transcription (STAT), suppressor of cytokine signaling 1 and 3 (SOCS1/3), and nuclear factor kappa‐light‐chain‐enhancer of activated B cells (NF‐κB) signaling pathways [20, 21]. TAMs are also able to inhibit inflammatory cytokine production and CD8^+^ T cell responses [22, 23, 24] by engulfing apoptotic cells via the process of efferocytosis, in which TAM receptors bind to phosphatidylserine on apoptotic cells [25, 26]. We found that NLRC5 deficiency enhances AXL and MERTK dependent efferocytosis and increases the expression and secretion of anti‐inflammatory mediators: interleukin‐10 (IL‐10), transforming growth factor‐beta (TGF‐β), *Socs1* and *Socs3*. Taken together, our findings provide the first evidence linking NLRC5 deficiency and TAM‐mediated efferocytosis, thereby providing new insights into the role of NLRC5 in antitumor immunity. We propose that increased TAM‐mediated efferocytosis creates an immunosuppressive environment that contributes to gastric B‐cell lymphoid neogenesis to *Helicobacter* infection in the *Nlrc5* ^mø‐KO^ mouse model.

## 2 Materials and Methods

### 2.1 Mice

Mice were maintained at the Monash Animal Research Platform, Monash Medical Centre, under specific pathogen‐free conditions. All animal experiments and procedures were approved by the Monash Medical Centre Animal Ethics Committee (approval MMCB/2020/42). The generation and characterization of *Nlrc5* ^fl/fl^ (wild type, WT) and *Nlrc5* ^mø‐KO^ C57BL/6 mice was described in detail elsewhere [10].

### 2.2 Bacteria

*H. pylori* Sydney Strain 1 (SS1) and *Helicobacter felis* (ATCC 49179/CS1) were routinely grown on blood agar medium or, for cell coculture experiments, in broth using methods described previously [27].

### 2.3 Animal Infection

WT and *Nlrc5* ^mø‐KO^ mice were orally gavaged with either Brain Heart Infusion broth (Thermo Fisher Scientific, Scoresby, VIC, Australia) or *H. felis* (equivalent to approximately 10^7^ colony‐forming units) [10, 11]. Animals were sacrificed at 12 weeks post‐gavage. Mouse splenic macrophages from control or infected mice were isolated and sorted by flow cytometry using F4/80^+^ CD45^+^ CD11b^+^ expression markers [11]. RNA was isolated from splenic macrophages using Quick‐RNA Microprep kit (Zymo Research).

### 2.4 Cell Lines

Human WT and *NLRC5*‐deficient (*NLRC5* ^−/−^) THP‐1 monocytes, generated by CRISPR‐Cas9 gene editing [10], and Jurkat cells were maintained at 37°C, 5% CO_2_ in complete Roswell Park Memorial Institute (RPMI) medium (cRPMI; Thermo Fisher Scientific), supplemented with 10% (v/v) fetal bovine serum (FBS), 2 mM 4‐(2‐hydroxyethyl)‐1‐piperazineethanesulfonic acid (HEPES) and 10 mM L‐glutamine (Thermo Fisher Scientific). Macrophages were derived from THP‐1‐monocytes by exposure to phorbol‐12‐myristate 13‐acetate (PMA; 20 ng/mL) for 48 h.

### 2.5 Isolation of Mouse Splenic and Bone Marrow‐Derived Macrophages (BMDMs)

For the isolation of splenic macrophages, spleens were harvested from both WT and *Nlrc5* ^mø‐KO^ mice and immediately placed in 10% (v/v) FBS in phosphate‐buffered saline (PBS) on ice. Spleens were minced, homogenized, and passed through cell strainers (70 μm). Following red blood cell lysis, F4/80^+^ splenic macrophages were isolated using anti‐F4/80‐Microbeads Ultrapure (Miltenyi Biotech, 130‐110‐443) according to the manufacturer's instructions. Isolated macrophages were then maintained in cRPMI containing 30% (v/v) L929‐cell‐conditioned medium. BMDMs were isolated by flushing the femurs and tibias of mice with 23‐gauge needles (BD Biosciences, San Jose, CA). Following collection, cells were centrifuged at 200 × g for 5 min, then resuspended in complete Dulbecco Modified Eagle's Medium (cDMEM) supplemented with 30% (v/v) L929‐cell‐conditioned medium [10]. BMDMs were subsequently cultured in low adherence flasks (Sarstedt, Numbrecht, Germany) and maintained at 37°C with 5% CO_2_.

### 2.6 Cell Coculture Assays

Splenic macrophages and BMDMs were seeded in 6‐(1 × 10^6^ cells/ml) or 24‐well plates (2.5 × 10^5^ cells/ml) and incubated at 37°C with 5% CO_2_. PMA‐treated WT and *NLRC5* ^−/−^ THP‐1 cells were seeded in 6‐ or 24‐well plates and incubated at 37°C with 5% CO_2_. Liquid cultures of *H. pylori* and *H. felis* were used for coculture assays at a multiplicity of infection (MOI) = 10, confirmed by viable counting [27]. As controls, cells were treated with known NLRC5 agonists, recombinant IFN‐γ (100 ng/mL; BioLegend, San Diego, CA) or *Escherichia coli* O111: B4 lipopolysaccharide (LPS, 100 ng/mL; UltraPure, InvivoGen, tlrl‐3pelps) [10].

### 2.7 Western Blotting

Protein lysates were extracted using Radioimmunoprecipitation assay (RIPA) buffer containing protease and phosphatase inhibitor cocktail (Roche, Switzerland). Cell extracts were resolved on SDS‐polyacrylamide gels (SDS‐PAGE) and transferred to polyvinylidene difluoride (PVDF) membrane. Membranes were blocked with 5% (w/v) bovine serum albumin (BSA) at room temperature for 1 h and subsequently incubated overnight at 4°C with the primary antibodies diluted in Tris‐buffered saline with 0.05% (v/v) Tween 20 (TBST) containing 3% (w/v) BSA. After washing, membranes were incubated with horseradish peroxidase (HRP) conjugated secondary antibodies and ECL SuperSignal West Pico Chemiluminescent Substrate (Thermo Fisher Scientific, RPN 2209). The primary Antibodies used for Western blotting are listed in Table S1.

### 2.8 Quantitative Polymerase Chain Reaction (qPCR)

Macrophages were seeded in 24‐well plates and stimulated with the indicated stimuli for 2 h, unless specified. RNA was extracted using Quick‐RNA MicroPrep, as per the manufacturer's instructions. RNA concentration and purity were assessed, and equal amounts of RNA were reverse transcribed into complementary DNA (cDNA) using random hexamer (1 μL), 10 mM dNTP mix (1 μL), 5× RT Buffer (4 μL), RiboSafe RNase Inhibitor (1 μL), Tetro Reverse Transcriptase (200 u/μL; 1 μL) (BIO‐65043; Meridian Bioscience). qPCR was performed in 10 μL reaction volumes, using the 2X‐SYBR Green PCR Master Mix (5 μL) (Thermo Fisher Scientific) and gene‐specific primers (0.5 μM; listed in Tables S2 and S3) on a QuantStudio 6 Flex Real‐Time PCR System (Applied Biosystems, MHTP Medical Genomics Facility). Cycling conditions consisted of an initial activation step at 95°C for 2 min, followed by 40 cycles of denaturation at 95°C for 15 s and annealing/extension at 60°C for 1 min. Melt curve analysis was performed at 95°C for 15 s, 60°C for 1 min, followed by a slow ramp (0.05°C/s) to 95°C for 15 s. Gene expression levels were quantified using the comparative Ct (ΔΔCt) method, and glyceraldehyde 3‐phosphate dehydrogenase (*GAPDH* or *Gapdh*) was used as an internal reference gene.

### 2.9 Cytokine ELISA

Mouse splenic macrophages were either left untreated or stimulated for 24 h with LPS (100 ng/mL), *H. pylori* , or *H. felis* bacteria (both at MOI = 10). Following stimulation, culture supernatants were collected for cytokine analysis. Cytokine concentrations were measured using kits, according to the manufacturer's instructions (IL‐6, BD Bioscience, 555240; IL‐10, BD Bioscience, 555240; TGF‐β, Thermo Fisher Scientific, 88‐8350‐22). Absorbance at 450 nm was recorded using a FLUOStar Optima microplate reader (BMG Labtech, Mornington, VIC, Australia), and cytokine concentrations were calculated using a four‐parameter logistic curve fit model.

### 2.10 Generation of Fluorescently Labeled Apoptotic Cells (ACs)

Mouse thymocytes and Jurkat cells were labeled with the fluorescent membrane dye DiO (Thermo Fisher Scientific, V22886), according to the manufacturer's instructions. Apoptosis was induced by treating the labeled cells with staurosporine (1 μM; Saphire Bioscience, ALX‐380‐014‐C100) for 4 h (thymocytes) or 3 h (Jurkat cells) at 37°C with 5% CO_2_. Apoptosis was assessed using the Annexin V Apoptosis Detection Kit with propidium iodide (PI) (Thermo Fisher Scientific, V13241). Flow cytometry was performed on a BD Fortessa instrument (MHTP Flowcore), and data were analyzed using FlowJo software (version 10.8, BD Life Sciences). At the specified time points, approximately 83% of cells displayed an early apoptotic phenotype (Annexin V^+^ PI^−^) (Figure S1).

### 2.11 Phagocytosis Assay

Cells were seeded at a density of 2.5 × 10^5^ cells/well (24‐well plates) or 1.25 × 10^5^ cells/well (48‐well plates). *H. pylori* bacteria were labeled with DiO and incubated with macrophage cultures (MOI = 10) in cRPMI medium for 2 h. To block bacterial internalization, macrophages were pretreated with cytochalasin D (5 μg/mL; Sigma, C8273‐1MG) for 30 min before and during coculture. After incubation, extracellular bacteria were removed by washing with ice‐cold PBS. Bacterial uptake was assessed by flow cytometry and expressed as both the percentage of DiO‐positive macrophages (phagocytosis %) and MFI.

### 2.12 Efferocytosis Assay

Efferocytosis was assessed by seeding at 2.5 × 10^5^ cells/well (24‐well plates) or 1.25 × 10^5^ cells/well (48‐well plates). For efferocytosis, DiO‐labeled apoptotic Jurkat cells were cocultured with THP‐1 macrophages at a 1:1 ratio, whereas DiO‐labeled apoptotic thymocytes were used with splenic macrophages and BMDMs (1:1 ratio). For selected conditions, macrophages were preincubated with recombinant human GAS6 (100 ng/mL; R&D Systems, 2636‐GS) for 30 min at 37°C in 5% CO_2_. For inhibition studies, macrophages were serum‐starved for 2 h prior to treatment for 2 h with MERTK or AXL inhibitors (both at 0.5 μM; UNC2025, HY‐12344A, or R428, HY15150, respectively; Focus Bioscience). After inhibitor removal, apoptotic cells were added in RPMI containing 10% (v/v) FBS and incubated for 1 h. To block ACs internalization, macrophages were pretreated with cytochalasin D, as described above. Efferocytosis was quantified by flow cytometry as the percentage of DiO‐positive macrophages and by measuring the mean fluorescence intensity (MFI) of internalized ACs.

### 2.13 Antigen Cross‐Presentation Assay

Thymocytes were isolated from transgenic OT‐1 mice expressing an H‐2K^b^‐restricted T cell receptor specific for the endotoxin‐free ovalbumin (OVA)_257–264_ peptide, SIINFEKL. The thymocytes (2.5 × 10^5^) were pulsed and loaded with this MHC class I‐restricted peptide (1 μM) for 2 h. SIINFEKL‐loaded apoptotic thymocytes were then generated by treatment with staurosporine (1 μM) for 4 h at 37°C in 5% CO_2_. Splenic macrophages (2.5 × 10^5^) isolated from WT or *Nlrc5* ^*mø*‐KO^ mice were cocultured with SIINFEKL‐loaded apoptotic cells for 2 h to facilitate efferocytosis. OT‐I T cells were labeled with carboxyfluorescein succinimidyl ester (CFSE, 5 μM; Invitrogen, C34554) using standard protocols. CFSE‐labeled OT‐I T cells (1 × 10^6^/well) were then cocultured with the macrophages for 72 h, as described previously [28]. Cells were stained with antibodies against CD4 (BD Bioscience, 553652), CD8α (BD Biosciences, 557654) and CD19 (Biolegend, 152 409) (Table S4). T cell proliferation was assessed by flow cytometry.

### 2.14 Statistical Analysis

Data analyses were performed using GraphPad‐Prism 10 (GraphPad Software LLC). Statistical significance was calculated using the Two‐way Analysis of Variance (ANOVA), One‐way ANOVA, or Mann–Whitney *U* test, as appropriate. If not stated otherwise, data were presented as mean ± SEM.

## 3 Results

### 3.1 TAM Receptor Expression Is Upregulated in Macrophages Lacking Functional NLRC5

**FIGURE 1:**
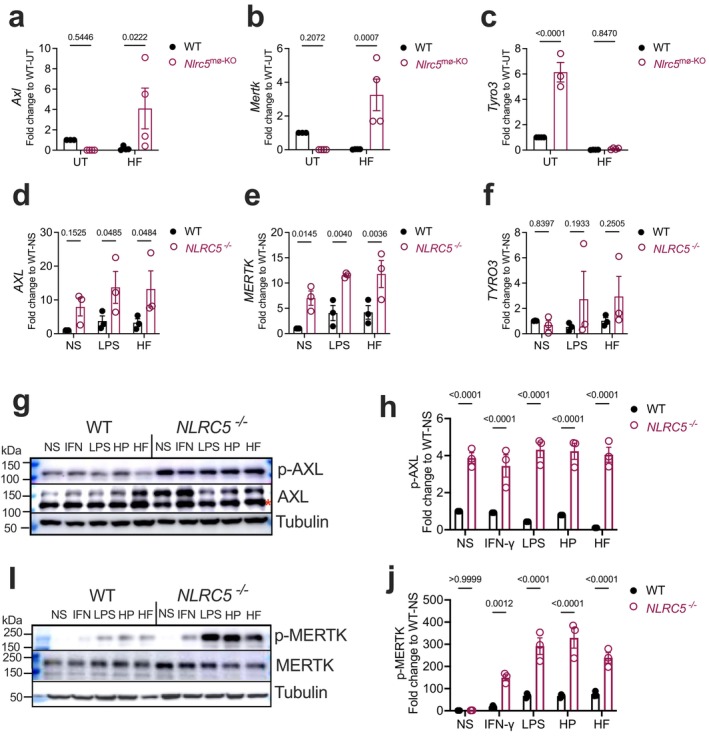
TAM receptor expression is upregulated in macrophages lacking functional NLRC5. Expression of TAM receptor genes (a) *Axl*, (b) *Mertk* and (c) *Tyro3* was detected in F4/80^+^ CD45^+^CD11b^+^ splenic macrophages from *H. felis* ‐infected WT and *Nlrc5* ^mø‐KO^ mice. Expression of TAM receptor genes (d) *AXL*, (e) *MERTK* and (f) *TYRO3* in WT and *NLRC5* ^−/−^ THP‐1 macrophages that were either not stimulated (NS) or stimulated with LPS (100 ng/mL) or *H. felis* (HF) bacteria (MOI = 10). Western blot detection of (g) total AXL and p‐AXL ^(Y702)^, (h) p‐AXL protein band intensity (i) total MERTK and p‐MERTK^(Tyr749/753/754)^, and (j) p‐MERTK protein band intensity in WT and *NLRC5* ^−/−^ THP‐1 macrophages that were either not stimulated (NS) or stimulated with IFN‐γ (100 ng/mL), LPS (100 ng/mL), *H. pylori* (HP) or *H. felis* (HF) bacteria (both MOI = 10), for 24 h. Gene expression was normalized to that of *Gapdh* or *GAPDH*, respectively. Tubulin was used as a loading control for Western blots. Densitometric assessment of band intensities was performed using ImageJ and normalized to tubulin. Data are presented as the mean ± SEM for triplicate determinations from (a–c) *n* = 3–4 mice/group or (d–f) *n* = 3 independent experiments. Data were analyzed by two‐way ANOVA. (g, h) Western blots are representative of *n* = 3 biological replicates.

NLRC5 is mainly known for its classical role as a regulator of MHC‐I antigen presentation and antitumor immunity [13, 14, 15], however, it has also been reported to have broad immune functions [10, 29]. Here, we investigated the role of NLRC5 in macrophage polarization and associated functions. Importantly, we found that splenic macrophages from *H. felis* ‐infected *Nlrc5* ^mø‐KO^ mice exhibited significantly elevated expression of TAM receptors, associated with immunosuppressive functions and known to promote cancer progression [17] (Figure 1). We showed that following stimulation with *H. felis* bacteria, expression levels for TAM receptor genes *Axl* (Figure 1a) and *Mertk* (Figure 1b) but not *Tyro3* (Figure 1c) were significantly upregulated in the macrophages from *H. felis* ‐infected *Nlrc5* ^mø‐KO^ mice, when compared with those from *H. felis* ‐infected WT animals (*p* < 0.05). Consistent with these findings, *NLRC5* ^−/−^ human THP‐1 macrophages displayed elevated gene expression of *AXL* (Figure 1d) and *MERTK* (Figure 1e) in response to stimulation with LPS or *H. felis* bacteria (*p* < 0.05), whereas *TYRO3* expression was not significantly increased when compared with WT cells (Figure 1f).

To demonstrate the activation of TAM signaling [30], we assessed phosphorylation of AXL and MERTK in *NLRC5* ^−/−^ and WT THP‐1 macrophages both constitutively and in response to stimulation. For this, we used: NLRC5 agonists, IFN‐γ and LPS; the human pathogen, *H. pylori* SS1; and *H. felis* , which induces the most severe pathology in the *Nlrc5* ^mø‐KO^ mouse model [10]. Interestingly, whereas AXL phosphorylation was constitutively increased in uninfected *NLRC5* ^−/−^ macrophages, when compared with WT cells (Figure 1g,h), MERTK phosphorylation was enhanced in response to stimulation with NLRC5 agonists or *Helicobacter* bacteria (Figure 1i,j). These results suggest that NLRC5 regulates basal TAM receptor expression and macrophage programming, but that expression of the individual receptors is selectively increased by microbial stimuli.

### 3.2 Phagocytic Activity Is Enhanced in Macrophages Lacking Functional NLRC5

**FIGURE 2:**
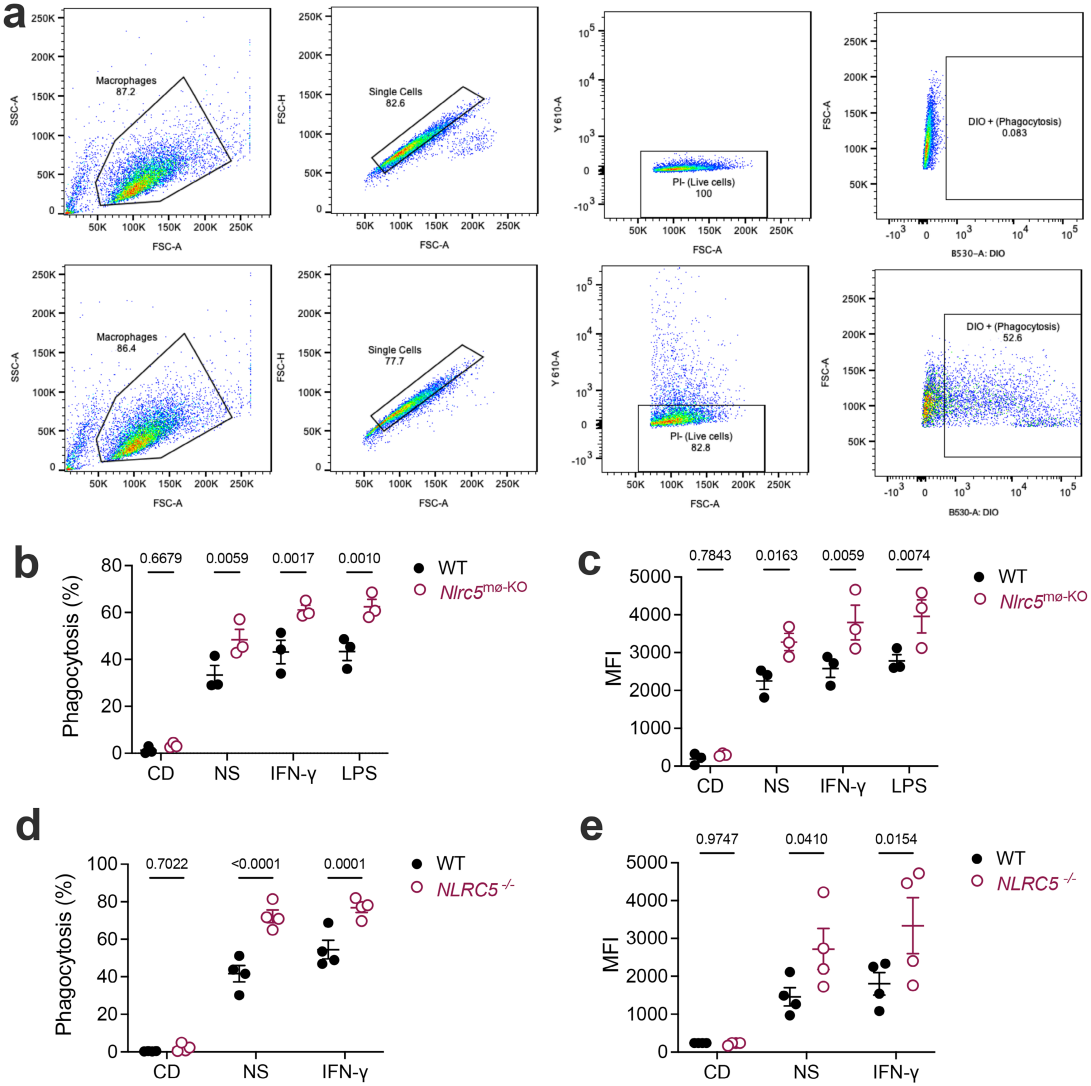
Phagocytic activity is enhanced in macrophages lacking functional NLRC5. Phagocytosis of DiO‐labeled *H. pylori* bacteria was measured by flow cytometry and expressed as % fluorescence‐positive cells (Phagocytosis %) or MFI. (a) Flow cytometry gating strategy: Negative control (top panel) and a representative sample (bottom panel) are shown. Phagocytosis (%) and MFI in (b, c) mouse splenic macrophages from WT and *Nlrc5* ^mø‐KO^ mice or (d, e) WT and *NLRC5* ^*−/−*^ THP‐1 macrophages. Macrophages were either not stimulated (NS) or stimulated with either IFN‐γ or LPS (both 100 ng/mL). Data are shown as the mean ± SEM from 3 to 4 independent experiments and analyzed by two‐way ANOVA. CD, Cytochalasin D; NS, non‐stimulated.

As one of the key functions of TAMs is phagocytosis [31], we next assessed the phagocytic activity of macrophages with defective Nlrc5 signaling. Interestingly, we found that mouse splenic macrophages from *Nlrc5* ^mø‐KO^ mice exhibited significantly higher levels of bacterial phagocytosis when compared with macrophages from WT animals, and this seemed to be independent of stimulation with either IFN‐γ or LPS (Figure 2a–c). Similar findings were found for human THP‐1 macrophages (Figure 2d,e). Enhanced levels of bacterial phagocytosis have been observed in M2 macrophages by other workers [32, 33]. Thus, the data are consistent with the suggestion that NLRC5 deficiency promotes the development in macrophages of a TAM M2 phenotype which is characterized functionally by enhanced bacterial phagocytosis.

### 3.3 Anti‐Inflammatory Responses Are Increased in Macrophages Lacking Functional Nlrc5

**FIGURE 3:**
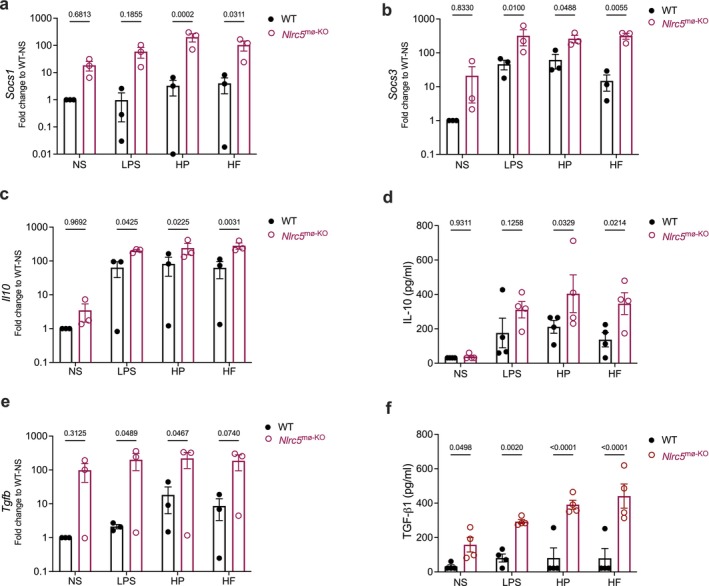
Anti‐inflammatory responses are increased in macrophages lacking functional Nlrc5. Expression of anti‐inflammatory factors (a) *Soc1*, (b) *Socs3*, (c) *Il10*, (d) IL‐10, (e) *Tgfb* and production of (f) TGF‐β1 in mouse splenic macrophages from WT and *Nlrc5* ^mø‐KO^ mice. Macrophages were either not‐stimulated (NS) or stimulated with LPS (100 ng/mL), *H. pylori* or *H. felis* bacteria (both MOI = 10) for 2 h. Gene expression was normalized to that of *Gapdh*. Cytokine concentrations in cell culture supernatants were determined by ELISA after 24 h. Data are the means ± SEM for triplicate determinations from *n* = 3–4 independent experiments and analyzed by two‐way ANOVA.

It was previously shown that TAM receptor kinases can mediate immunosuppression via induction of two suppressors of cytokine signaling, SOCS1 and SOCS3, which act on the JAK–STAT pathway to negatively regulate TLR responses [20]. Interestingly, *Socs1* and *Socs3* expression was significantly upregulated in *Nlrc5* ^−/−^ splenic macrophages stimulated with LPS or *Helicobacter* bacteria, when compared with WT macrophages (Figure 3a,b, respectively; *p* < 0.05). Furthermore, we also observed significantly higher levels of gene expression and protein for the anti‐inflammatory cytokines, IL‐10 and TGF‐β, in *Nlrc5* ^−/−^ macrophages when compared with WT cells (Figure 3c–f) (*p* < 0.05). This observation is consistent with TAMs, which are known to have elevated IL‐10 and TGF‐β production [16, 34]. In contrast, we observed no significant differences between WT and *Nlrc5* ^−/−^ macrophages for the expression of pro‐inflammatory cytokine genes encoding IL‐1β, IL‐6, interferon‐β (IFN‐β) or tumor necrosis factor (TNF) (Figure S2). *Nlrc5* deficiency appears to promote a skewed immunosuppressive program characterized by increased IL‐10 and TGF‐β expression without a concomitant reduction or significant alteration in canonical pro‐inflammatory cytokines, such as IL‐1β, IL‐6 or TNF. This is consistent with emerging evidence suggesting that TAMs exist along a dynamic phenotypic spectrum rather than as discrete M1 or M2 populations, with mixed inflammatory and immunoregulatory features commonly observed within the tumor microenvironment [35, 36, 37]. Altogether, these findings suggest that defective NLRC5 signaling in macrophages promotes anti‐inflammatory responses that we propose leads to an immunosuppressive milieu which drives tumor progression.

### 3.4 Increased Phosphorylation of STAT3 but Not STAT1 or STAT5 in Macrophages Lacking Functional NLRC5

**FIGURE 4:**
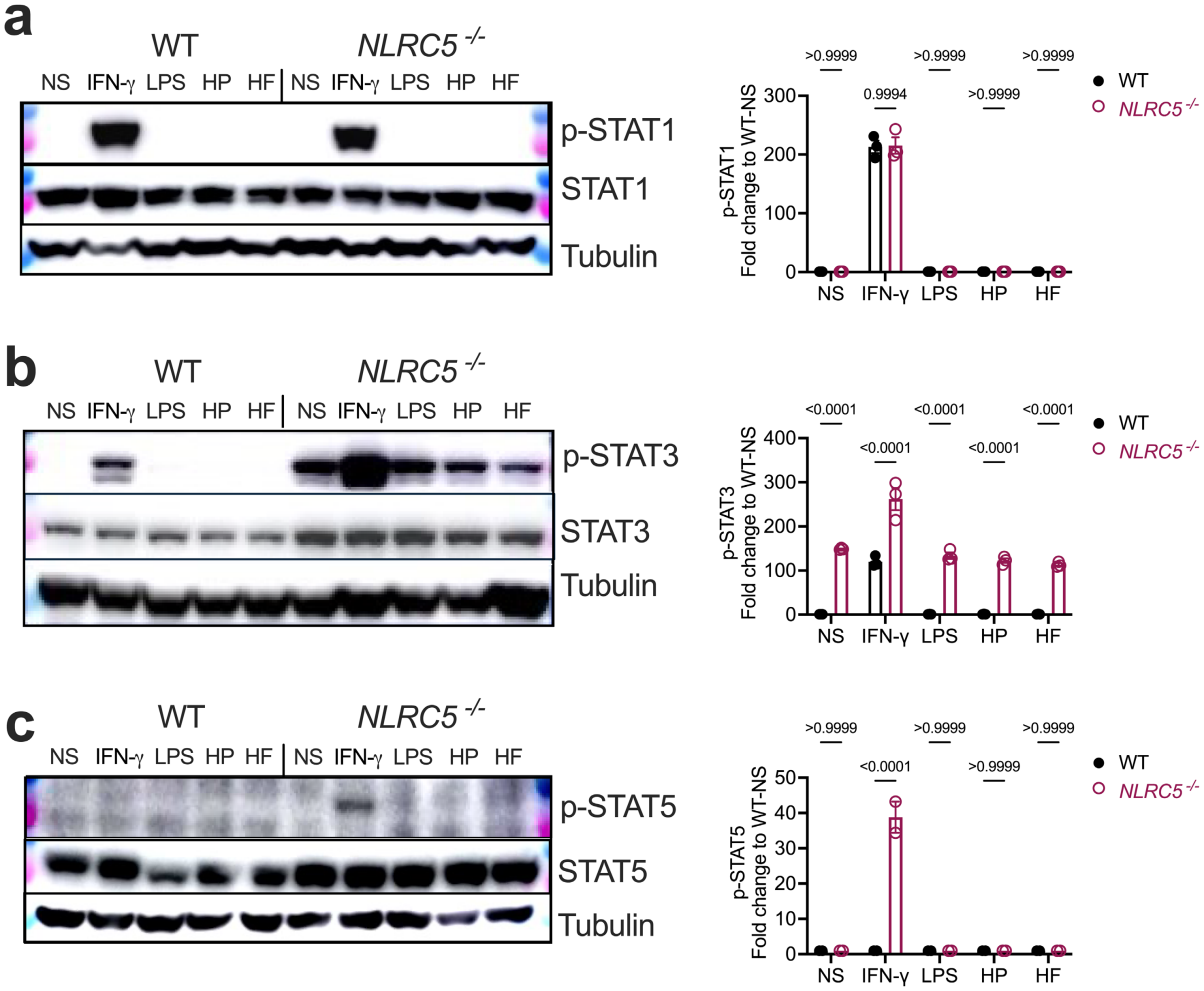
Increased phosphorylation of STAT3 but not STAT1 or STAT5 in macrophages lacking functional NLRC5. Western blot detection of total and phosphorylated forms of (a) STAT1^(Tyr701)^, (b) STAT3^(Tyr705)^ and (c) p‐STAT5^(Tyr694)^, and their corresponding band intensities, in WT and *NLRC5* ^*−/−*^ THP‐1 macrophages. Cells were either not‐stimulated (NS) or stimulated with IFN‐γ (100 ng/mL), LPS (100 ng/mL), *H. pylori* (HP) or *H. felis* (HF) (both MOI = 10) for 2 h. Densitometric assessment of band intensities was performed using ImageJ and normalized to tubulin. Representative Western blot images are shown for *n* = 3 independent experiments.

It was reported that TAMs can inhibit TLR‐induced inflammatory responses via an IFNAR/STAT1 pathway triggered by signaling via the receptors, AXL and MERTK [20]. TAM receptors can also form a positive feedback loop with STAT3 activation, which induces immunosuppressive cytokine responses (e.g., IL‐6, IL‐10, and TGF‐β) leading to a positive feedback loop that amplifies STAT3 signaling and supports tumor growth and progression [38, 39]. To investigate the mechanism by which NLRC5 regulates TAM‐mediated suppression of inflammatory responses, we studied STAT phosphorylation in WT and *NLRC5* ^−/−^ THP‐1 macrophages that were stimulated with IFN‐γ, LPS, *H. pylori* or *H. felis* bacteria for 30 min to 4 h (Figure 4, Figure S3). Phosphorylation of STAT1 (p‐STAT1^Y701^) was induced in response to IFN‐γ at similar levels in WT and *NLRC5* ^−/−^ THP‐1 macrophages but not following stimulation with either LPS or *Helicobacter* spp. (Figure 4a, Figure S3a). In contrast, we observed an increase in phosphorylated STAT3 (p‐STAT3^Y705^) in *NLRC5* ^−/−^ THP‐1 macrophages compared with WT cells to stimulation with IFN‐γ (Figure 4b, Figure S3b), which is consistent with reports indicating that p‐STAT3 is a downstream effector of TAM receptor kinase signaling [21]. p‐STAT3^Y705^ levels were also increased in *NLRC5* ^−/−^ macrophages stimulated with LPS or *Helicobacter* spp., but the levels did not vary from those in unstimulated cells (Figure 4b, Figure S3b). Like for STAT1, STAT5 phosphorylation was only detected in response to IFN‐γ stimulation and did not differ between the cell lines (Figure 4c, Figure S3c). Collectively, these findings demonstrate that STAT3 but not STAT1 or STAT5 is constitutively upregulated in *NLRC5* ^−/−^ macrophages, a finding that is consistent with their TAM phenotype and suggestive of a role for STAT3 in the autocrine or paracrine induction of immunosuppressive cytokine responses. Conversely, STAT3 is unlikely to be involved in regulation of immunosuppressive cytokine responses to *Helicobacter* bacteria.

### 3.5 Increased Efferocytosis of Apoptotic T Cells in Macrophages Lacking Functional Nlrc5

**FIGURE 5:**
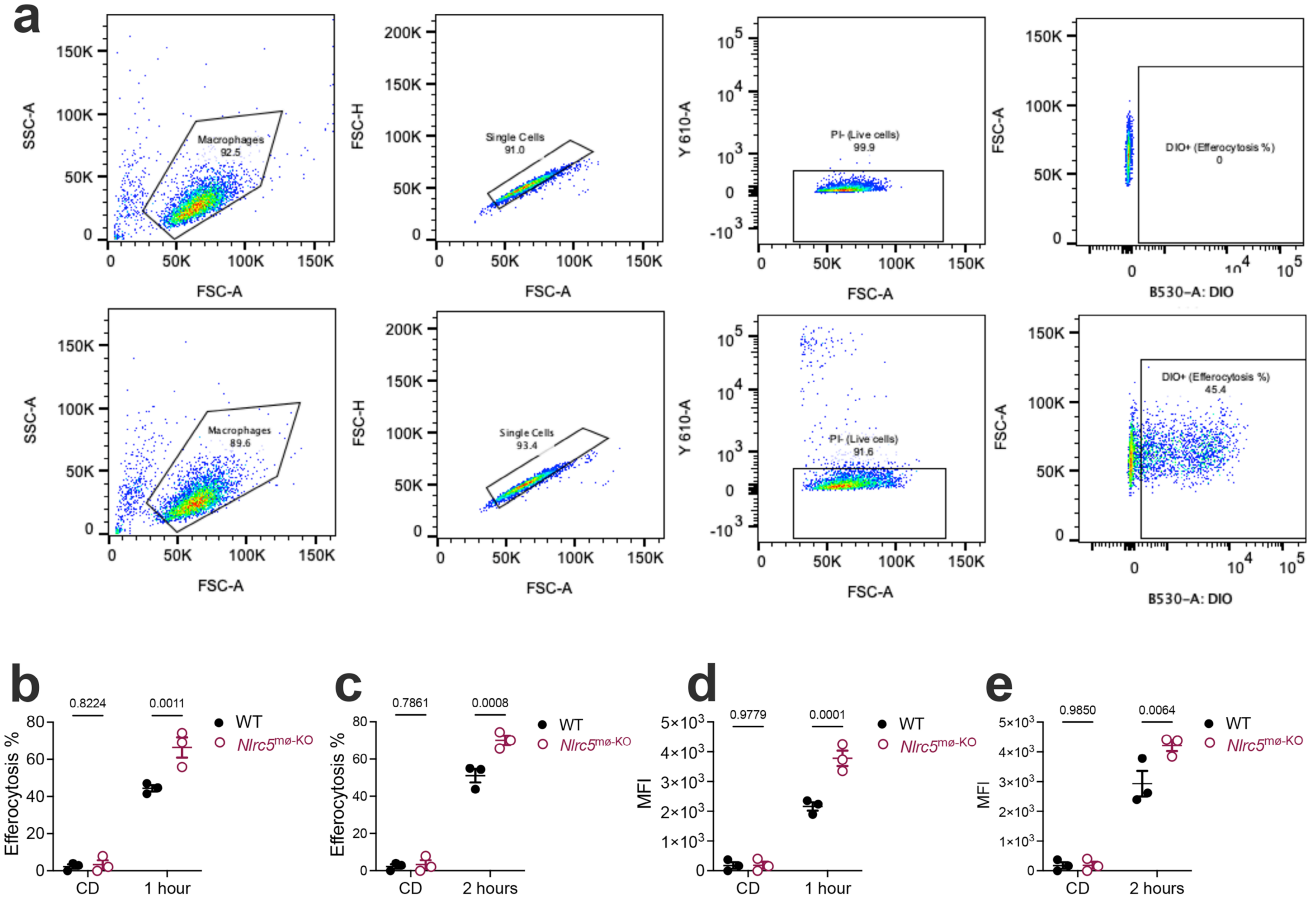
Increased efferocytosis of apoptotic T cells in macrophages lacking functional Nlrc5. Splenic macrophages from WT or *Nlrc5* ^mø‐KO^ mice were cocultured with DiO‐labeled apoptotic mouse thymocytes (1:1 ratio). (a) Flow cytometry gating strategy. Efferocytosis was measured by flow cytometry and expressed as (b, c) the percentage of DiO^+^ cells (efferocytosis %) or (d, e) MFI at (b, d) 1 or (c, e) 2 h. Data are presented as the means ± SEM for triplicate determinations from *n* = 3 biological replicates and analyzed by the Mann–Whitney *U* test.

Efferocytosis is important in mediating inflammation resolution and promoting antitumor immunity [25, 26]. Given that efferocytosis is promoted by TAM signaling [25, 26], and that TAM receptor expression was upregulated in *Nlrc5*/*NLRC5* KO macrophages (Figure 1), we sought to determine whether efferocytosis may contribute to immunosuppressive responses mediated by defective NLRC5 signaling in macrophages. For this, we determined macrophage uptake of DiO‐labeled apoptotic mouse thymocytes or Jurkat cells by flow cytometry (Figure 5 and Figure S4). We demonstrated significantly higher efferocytosis efficiency in *Nlrc5* ^−/−^ mouse splenic macrophages when compared with WT macrophages at 1‐ and 2‐h coculture with DiO^+^ apoptotic thymocytes (Figure 5b,c; *p* < 0.0011 and *p* < 0.0008, respectively). The MFI of macrophages having engulfed DiO^+^ apoptotic thymocytes was also approximately two‐fold higher in *Nlrc5* ^−/−^ macrophages compared with WT cells (Figure 5d,e; *p* < 0.0001 and *p* < 0.0064, respectively). Similarly, *NLRC5* ^−/−^ THP‐1 macrophages were significantly more efficient in engulfing apoptotic Jurkat cells when compared with WT THP‐1 macrophages (Figure S4b–e), which is consistent with previous work showing enhanced efferocytosis in M2‐polarized THP‐1 macrophages [40].

To determine whether the enhanced efferocytosis observed in *Nlrc5*/*NLRC5* KO macrophages was cell‐type specific, we assessed efferocytosis by BMDMs isolated from WT and *Nlrc5* ^mø‐KO^ mice. Importantly, however, no significant differences in efferocytosis efficiency were observed between these BMDMs, nor was efferocytosis increased in response to stimulation with NLRC5 agonists, IFN‐γ or LPS (Figure S5). This can be explained by the fact that splenic macrophages develop within a specialized niche [41, 42, 43], whereas BMDMs are monocyte‐derived macrophages generated in vitro in the presence of growth factors and therefore incompletely reproduce the identity of tissue‐resident macrophages [41]. Indeed, substantial phenotypic and functional differences have been observed between splenic macrophages and BMDMs [41], with differences in M1/M2 polarization bias and metabolic programming further contributing to their functional divergence [41, 44].

Collectively, these findings indicate that defective Nlrc5 signaling in splenic macrophages promotes a TAM phenotype (Figure 1), which enhances efferocytosis efficiency and that, ultimately, we propose, contributes to an immunosuppressive state which is conducive to the gastric B‐cell lymphoid neogenesis observed in the *Nlrc5* ^mø‐KO^ mouse model [10, 11, 12].

### 3.6 TAM Receptors Promote Enhanced Efferocytosis in Macrophages Lacking Functional NLRC5

**FIGURE 6:**
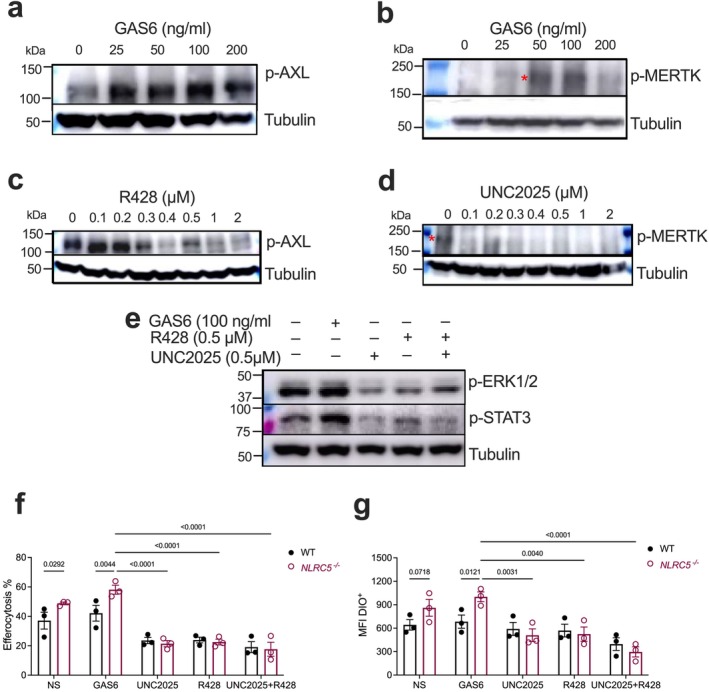
TAM receptors promote enhanced efferocytosis in macrophages lacking functional NLRC5. Western blot analysis of (a, b) *NLRC5* ^−/−^ THP‐1 macrophages treated with increasing concentrations (0–200 ng/mL) of the TAM ligand GAS6 (100 ng/mL, 30 min) and tested for the presence of phosphorylated (a) AXL (p‐AXL) or (b) MERTK (p‐MERTK) or R428/UNC2025 for 2 h. Phosphorylation of (a, c) AXL and (b, d) MERTK. *NLRC5* ^−/−^ THP‐1 macrophages were treated with either GAS6 (100 ng/mL, 30 min) or R428 and/or UNC2025 (0.5 μM, 2 h). Western blotting was used to detect downstream signaling molecules (e) p‐ERK1/2 and p‐STAT3. Tubulin was used as a loading control. (f, g) Flow cytometric analysis of efferocytosis of apoptotic Jurkat cells by WT and *NLRC5* ^*−/−*^ THP‐1 macrophages treated with either GAS6 (100 ng/mL, 30 min) or R428 and/or UNC2025 (0.5 μM, 2 h). Data are presented as the means ± SEM for triplicate determinations from *n* = 3 individual experiments and analyzed by two‐way ANOVA.

Enhanced expression and phosphorylation of the TAM receptors, AXL and MERTK, were observed in macrophages with defective NLRC5 signaling in response to inflammatory and bacterial stimuli (Figure 1). This finding was confirmed as the addition of the TAM ligand, GAS6, potentiated phosphorylation of AXL and MERTK in *NLRC5* ^−/−^ THP‐1 macrophages (Figure 6a,b, respectively). To dissect the contribution of AXL and MERTK in efferocytosis, we assessed their phosphorylation in response to GAS6 stimulation following pretreatment of macrophages with the small molecule inhibitors R428 [45] and UNC2025 [46], respectively (Figure 6c,d). We showed that these inhibitors could reduce AXL and MERTK phosphorylation in a dose‐dependent manner (Figure 6c,d), at concentrations with ≤ 5% cytotoxicity (Figure S6).

To further assess the effect of these inhibitors on TAM receptor signaling in *NLRC5* ^−/−^ THP‐1 macrophages, we determined phosphorylation of two of the known downstream signaling molecules, extracellular signal‐regulated kinase 1 and 2 (ERK 1/2) and STAT3 [47]. We showed that pretreatment with R428 or UNC2025 resulted in reduced levels of STAT3 and ERK1/2 phosphorylation in cells (Figure 6e). Importantly, we also demonstrated that inhibition of AXL or MERTK significantly reduced the efferocytosis efficiency of GAS6‐treated *NLRC5* ^−/−^ macrophages, when compared with untreated macrophages (Figure 6f,g). We did not, however, observe a notable additive effect when both inhibitors were used (Figure 6f,g), suggesting possible functional redundancy or involvement of overlapping pathways [22]. These data show that in the absence of NLRC5 signaling, the TAM receptor kinases AXL and MERTK are upregulated, thereby triggering a signaling cascade involving ERK1/2 and STAT3 that leads to enhanced efferocytosis.

### 3.7 MHC‐I‐Restricted Antigen Presentation Is Reduced in Macrophages Lacking Functional Nlrc5

**FIGURE 7:**
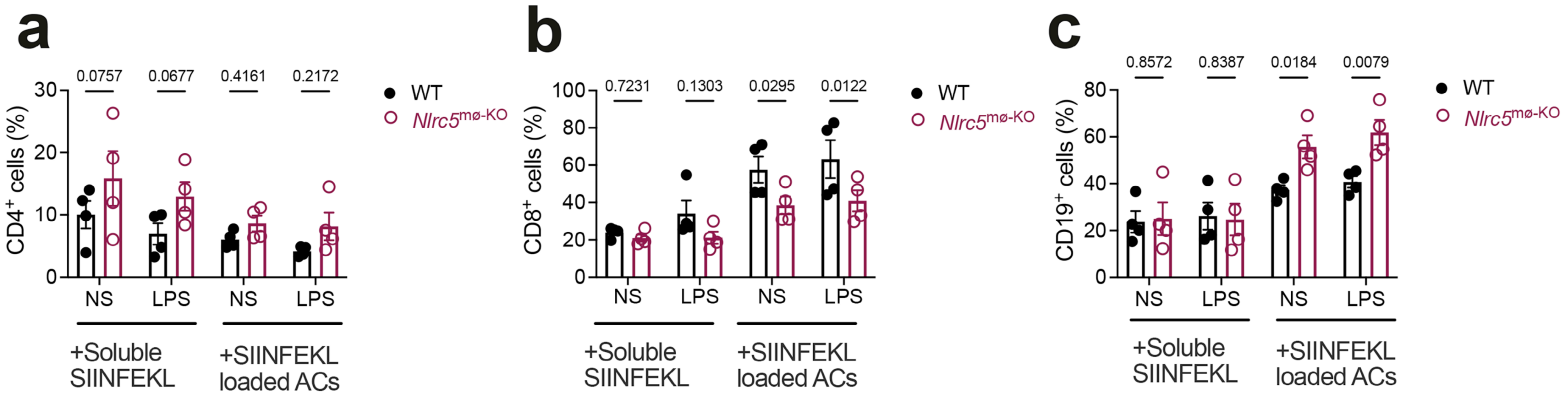
MHC‐I‐restricted antigen presentation is reduced in macrophages lacking functional Nlrc5. Flow cytometry was used to assess proliferation (expressed as a %) by (a) CD4^+^ T cells, (b) CD8^+^ T cells, and (c) CD19^+^ B cells that had been cocultured with splenic macrophages from WT or *Nlrc5* ^mø‐KO^ mice. Macrophages that had been either left unstimulated (NS) or pre‐stimulated with LPS (100 ng/mL) were cocultured with either soluble SIINFEKL or SIINFEKL‐loaded apoptotic mouse thymocytes, then co‐incubated with OT‐I cells for 72 h. Data are presented as the means ± SEM for triplicate determinations from *n* = 3 individual experiments and analyzed by two‐way ANOVA.

TAM‐mediated efferocytosis is important in restricting CD8^+^ T cell responses, which are essential for antitumor immune responses [24]. To determine the impact of Nlrc5 deficiency on antigen presentation to CD8^+^ T cells, we assessed the efferocytosis of apoptotic thymocytes by mouse splenic macrophages from WT and *Nlrc5* ^mø‐KO^ mice. For this, splenic macrophages from WT or *Nlrc5* ^mø‐KO^ mice were incubated with the MHC‐I‐restricted peptide SIINFEKL, either in soluble form or loaded onto apoptotic thymocytes, then cocultured with CFSE‐labeled OT‐I cells. We observed an increased but not significantly different trend in cell proliferation for CD4^+^ T cells cocultured with *Nlrc5* ^−/−^ macrophages, when compared with those exposed to WT macrophages (Figure 7a). Importantly, CD8^+^ T cell proliferation was significantly decreased in the presence of *Nlrc5* ^−/−^ macrophages, both constitutively and in response to LPS stimulation; however, this was only observed in the presence of SIINFEKL‐loaded apoptotic thymocytes (Figure 7b; *p* < 0.02). Consistent with the B‐cell hyperplasia in *Nlrc5* ^mø‐KO^ mice [10, 11, 12], we observed increased B‐cell proliferation for *Nlrc5* ^−/−^ but not WT macrophages that had been cocultured with SIINFEKL‐loaded apoptotic thymocytes and stimulated or not with LPS (Figure 7c; *p* < 0.02). These data show that although Nlrc5 deficiency promotes efferocytosis, macrophages lacking functional Nlrc5 are affected in their ability to perform MHC‐I‐restricted antigen presentation to CD8^+^T cells and are thus less able to participate in antitumor immunity.

## 4 Discussion

The innate immune protein NLRC5 is a positive regulator of MHC class I gene expression and mediator of CD8^+^ T cell responses required for antitumor immunity [15, 48]. In this study, we extend knowledge on the role of NLRC5 in antitumor immunity by revealing that NLRC5 is important in preventing the functional polarization of macrophages to the M2‐like phenotype of TAMs.

TAMs are immunosuppressive cells that arise from tissue‐resident or monocyte‐derived macrophages and migrate to tumor sites, thereby facilitating immune evasion and tumor progression [19]. We show that NLRC5‐deficient macrophages have the phenotypic and functional properties of TAMs, as characterized by increased expression of TAM receptor kinases, together with enhanced phagocytosis, efferocytosis, and anti‐inflammatory mediator expression, resulting in impaired antigen presentation to CD8^+^T cells. Furthermore, we identify STAT3 and ERK1/2 as being downstream targets of TAM receptor kinase activation in NLRC5‐deficient macrophages. In summary, we propose that defective NLRC5 signaling reprograms macrophages to a tumor‐permissive state thereby impacting a model of *Helicobacter*‐induced lymphomagenesis [10, 11, 12].

TAM kinases are associated with an M2 phenotype and are involved in efferocytosis, inflammation resolution and tissue repair [49, 50]. In *Nlrc5*/*NLRC5* KO macrophages, we observed selective upregulation of Axl/AXL and Mertk/MERTK, but not Tyro3/TYRO3 responses (Figure 1). This finding was consistent with previous reports showing that individual TAM receptors exhibit specificity for different stimuli and play distinct roles in phagocytosis [49]. *Nlrc5*/*NLRC5* KO macrophages displayed increased levels of phagocytosis (Figure 2), a characteristic of M2 macrophages [32, 33], suggesting that loss of functional NLRC5 promotes M2‐like TAM polarization. These findings are consistent with studies in which *NLRC5* overexpression was shown to suppress M2 polarization [51], whereas its silencing promoted M2 polarization [52].

The ability to perform efferocytosis is a key function of TAMs and contributes to immunosuppression through cytokine secretion and upregulation of the checkpoint inhibitor proteins, PD‐1/PD‐L1 [16, 17, 22, 24]. NLRC5‐deficient macrophages were shown to have enhanced efferocytosis, though this did appear to be cell‐specific, as efferocytosis was unaffected in the BMDMs from *Nlrc5* ^mø‐KO^ mice (Figure S5). By pharmacological inhibition, we showed that the enhanced efferocytosis in NLRC5‐deficient macrophages was dependent on the activity of the TAM kinases, AXL and MERTK (Figure 6).

Consistent with a TAM phenotype, Nlrc5‐deficient macrophages had higher gene expression levels for the suppressors of cytokines, Socs1 and Socs3, and secreted elevated amounts of cytokines that suppress inflammation and impair CD8^+^ T cell responses (Figure 3), notably IL‐10 and TGF‐β [16, 23, 35]. Interestingly, IL‐10 can activate STAT3, leading to PD‐1/PD‐L1 expression and M2 polarization [53, 54]. Furthermore, constitutive STAT3 activity has been associated with tumor immune evasion [21, 38, 39] and poor patient survival in diffuse large B‐cell lymphoma [55, 56]. Taken together, we propose that NLRC5 restrains TAM‐mediated immunosuppression in *Helicobacter* infection via STAT3 regulation and independently of STAT1 (Figure 4). The NLRC5‐STAT3 axis may thus represent a key regulatory pathway controlling TAM‐driven immunosuppression in this model; however, further investigation is required to demonstrate a mechanistic link between NLRC5 and STAT3. It is noteworthy that TAM‐mediated efferocytosis in this model required both AXL and MERTK phosphorylation (Figure 6), whereas the IFNAR‐STAT1 signaling axis [20] was reported to be dependent on AXL alone.

Previous studies reported that *Nlrc5* ^−/−^ mice exhibit impaired levels of MHC class I gene expression and reduced IFN‐γ and CD8^+^ T cell responses [14, 15, 57, 58]. Here, we also show that *Nlrc5* deficiency negatively impacted MHC‐I‐restricted CD8^+^ T cell activation (Figure 7b). A more interesting observation from the current work, however, was that Nlrc5‐deficient macrophages seemed to promote CD4^+^ T and CD19^+^ B‐cell proliferation (Figure 7a,c). This was particularly evident for CD19^+^ B‐cell proliferation, which was significantly increased in response to Nlrc5‐deficient macrophages loaded with MHC class I‐restricted peptide (Figure 7c). This shift away from cytotoxic CD8^+^ T‐cell activation and toward the immunosuppressive axis of TAMs is likely to be important in *Helicobacter*‐induced B‐cell lymphomagenesis, as TAMs secrete greater amounts of the survival factors required for B‐cell activation and proliferation [18, 59]. Indeed, we [10, 11, 12] and others [18, 60] have identified B‐cell survival factors to be critical for the formation of gastric MALT lymphoma induced by chronic *Helicobacter* infection.

Although our findings demonstrate that NLRC5 deficiency is associated with increased TAM receptor expression and enhanced immunosuppressive functions, additional mechanistic studies are required to further substantiate these observations. In particular, NLRC5 overexpression experiments would provide important complementary evidence by determining whether restoration of NLRC5 can reverse TAM receptor upregulation and the associated immunosuppressive phenotype. In addition, although the proposed role here for NLRC5 in tumor‐associated immune modulation is supported by our data from the MALT lymphoma model in mice [11, 12], it would be informative to assess the cytotoxic properties of infiltrating CD8^+^ T cells in gastric lesions and their role in progression of the disease over time in vivo. Likewise, it would be important to validate the findings using patient‐derived samples, however, MALT lymphoma is a rare cancer (i.e., an incidence of fewer than 15 per 100 000 people/year) and hence obtaining clinical material from cases of the disease is difficult. Additionally, it is important to note that the *Helicobacter* strains used here, *H. pylori* SS1 and *H. felis* , lack functional type IV secretion systems which are important for bacterial virulence [27]. Previous studies also showed that bacteria lacking this virulence factor were able to induce gastric B‐cell lymphoid neogenesis in mouse models [8, 9, 10, 11].

In conclusion, our findings position NLRC5 as a critical negative regulator of TAM‐associated gene expression, efferocytosis and immunosuppressive signaling. We propose that NLRC5 deficiency reprograms macrophages toward an M2‐like phenotype that promotes immune evasion and tumor progression. These results reveal a novel role for TAMs in *Helicobacter*‐mediated MALT lymphoma. As TAM receptor kinases are upregulated in inflammation‐driven lymphoma [18, 59, 61, 62, 63], these proteins have emerged as new potential targets in cancer immunotherapy [17]. We suggest that the targeting of TAMs and their receptors may therefore offer new therapeutic strategies for the treatment of gastric MALT lymphoma to *H. pylori* infection.

## Author Contributions

**Shambel Araya Haile:** conceptualization (supporting), investigation (lead), data curation (Lead), formal analysis (lead), writing – original draft (lead), writing – review and editing (supporting). **Alessandra Gatta:** investigation (supporting), data curation (supporting), formal analysis (supporting). **Nina Colon:** investigation (supporting). **Dana J. Philpott:** funding acquisition (lead), resources (supporting), expertise (supporting). **Thomas A. Kufer:** funding acquisition (lead), resources (supporting), expertise (supporting). **Le Ying:** conceptualization (supporting), investigation (supporting), funding acquisition (supporting). **Dongmei Tong:** conceptualization (supporting), data curation (supporting), formal analysis (supporting), investigation (supporting), supervision (supporting). **Richard L. Ferrero:** conceptualization (lead), funding acquisition (lead), project administration (lead), supervision (lead), investigation (supporting), formal analysis (supporting), writing – original draft (supporting), writing – review and editing (lead).

## Funding

Research in the RLF laboratory was funded by the US Department of Defense (Award No. W81XWH‐17‐1‐0606), Tour de Cure (Sydney, Australia; RSP‐330‐2020), and National Health and Medical Research Council (Project Grant APP1107930, Ideas Grant APP2012620, Senior Research Fellowship, APP1079904). Funding for the NLRC5 project was also provided to TAK and RLF from the DAAD‐Australia‐Germany Joint Research Cooperation Scheme. Shambel Araya Haile received a Monash International Tuition Scholarship (MITS) and Monash Graduate Scholarship (MGS), awarded by the Faculty of Medicine, Nursing and Health Sciences at Monash University. Alessandra Gatta was the recipient of an Erasmus IKA mobility grant from the European Union. Development of the *Nlrc5* ^fl/fl^ mice received partial funding from the Public Health Agency of Canada. Research conducted at the Hudson Institute of Medical Research is supported by the Victorian Government Operational Infrastructure Support Program.

## Conflicts of Interest

The authors declare no conflicts of interest.

## Supporting information


**Figure S1:** Generation of apoptotic cells. Thymocytes from wild type mice or human lymphocyte Jurkat cells were treated with 1 μM staurosporine for 2, 3, or 4 h to induce apoptosis. (a) Flowcytometry gating strategy; (b) Percentage of apoptotic (Red), Necrotic (brown) and viable (black line) thymocytes and (c) apoptotic Jurkat cells are shown.

**Figure S2:** Pro‐inflammatory cytokine responses are not significantly increased in *Nlrc5* ^−/−^ macrophages. Expression of (a) *Ifnb*, (b) *Il1b*, (c) *Il6*, and (d) *Tnf* in mouse splenic macrophages from WT and *Nlrc5* ^mø‐KO^ mice. Gene expression was normalized to that of *Gapdh*. Cells were either not‐stimulated (NS) or stimulated with either LPS (100 ng/mL), *H. pylori* (HP), *H. felis* (HF) (both MOI = 10) for 2 h. Data are presented as the means ± SEM for triplicate determinations from *n* = 3–4 biological replicates and analyzed by two‐way ANOVA.

**Figure S3:** Increased phosphorylation of STAT3 but not STAT1 or STAT5 in macrophages lacking functional NLRC5. Total and phosphorylated forms of (a) STAT1^(Tyr701)^, (b) STAT3^(Tyr705)^, and (c) p‐STAT5^(Tyr694)^ were detected by Western blotting in WT and *NLRC5* ^*−/−*^ THP‐1 macrophages. Cells were either not‐stimulated (NS) or stimulated with IFN‐γ (100 ng/mL), LPS (100 ng/mL), *H. pylori* (HP) or *H. felis* (HF) (both MOI = 10) for 30 or 60 min, or for 2 or 4 h. Representative Western blot images are shown for *n* = 3 independent experiments.

**Figure S4:** Increased efferocytosis of apoptotic T cells in macrophages lacking functional NLRC5. WT or *NLRC5* ^−/−^ THP‐1 macrophages were cocultured with DiO‐labeled apoptotic Jurkat cells (1:1 ratio). (a) Flow cytometry gating strategy. Efferocytosis was measured by flow cytometry and expressed as (b, c) the percentage of DiO^+^ cells (efferocytosis %) or (d, e) MFI at 30 min or 1 h. Data are presented as the means ± SEM for triplicate determinations from *n* = 4 biological replicates and analyzed by the Mann–Whitney *U* test.

**Figure S5:** Functional Nlrc5 deficiency does not result in increased efferocytosis or phagocytosis in mouse BMDMs. Bacterial phagocytosis and efferocytosis was measured by flow cytometry. (a, b) BMDMs from WT or *Nlrc5* ^mø‐KO^ mice were cocultured with DiO‐labeled apoptotic mouse thymocytes (1:1 ratio) and expressed as efferocytosis % and MFI at 1‐ or 2‐h. (c, d) BMDMs from WT or *Nlrc5* ^mø‐KO^ mice were cocultured with DiO‐labeled *H. pylori* (MOI = 10) and expressed as bacterial phagocytosis % and MFI in either not‐stimulated (NS) or stimulated with either IFN‐γ or LPS (both 100 ng/mL). Data are shown as the mean ± SEM and the presented values are combined from 3 independent experiments. Two‐way ANOVA. CD, Cytochalasin D.

**Figure S6:** Cytotoxicity level of the small molecules R428 and UNC2025. THP‐1 macrophages were left untreated (UT) or treated with either R428 or UNC2025 for 2 h at the indicated concentrations. Lactate dehydrogenase (LDH) released into the culture media was determined using a Cytotoxicity Detection Kit (Promega, G1780). Cytotoxicity level of R428 (Red line) and UNC2025 (Black line). PC, positive control.

**Figure S7:** MHC‐I‐restricted antigen presentation is reduced in macrophages lacking functional Nlrc5. Macrophages that had been either left unstimulated (NS) or pre‐stimulated with LPS (100 ng/mL) were cocultured with either soluble SIINFEKL or SIINFEKL‐loaded apoptotic mouse thymocytes, then co‐incubated with CFSE labeled OT‐I cells for 72 h. (a) Gating of CD4^+^ T cells and CD8^+^ T cells. (b) Percentages of CD8^+^ T cells that had undergone cell division, as observed by a reduction in CFSE staining. (c) Percentages of CD19^+^ B cells that had been cocultured with splenic macrophages from WT or *Nlrc5* ^mø‐KO^ mice. Representative images from three independent experiments.

**Table S1:** Antibody information for Western blotting.
**Table S2:** Primer sequences used for qPCR analysis (Human).
**Table S3:** Primer sequences used for qPCR analysis (Mouse).
**Table S4:** Antibody information for flowcytometry analyses.

## Data Availability

All relevant data supporting this study are provided within the manuscript and its Supporting Information.
