# Supplementary figures and images for: NLRC5‐Deficient Macrophages Promote a Tumor‐Permissive Phenotype via AXL‐ and MERTK‐Mediated Efferocytosis
Source: FASEB J. 2026 Aug 1;40(15):e72156. doi: 10.1096/fj.202504879R (PMC13428295; doi:10.1096/fj.202504879R)

**a**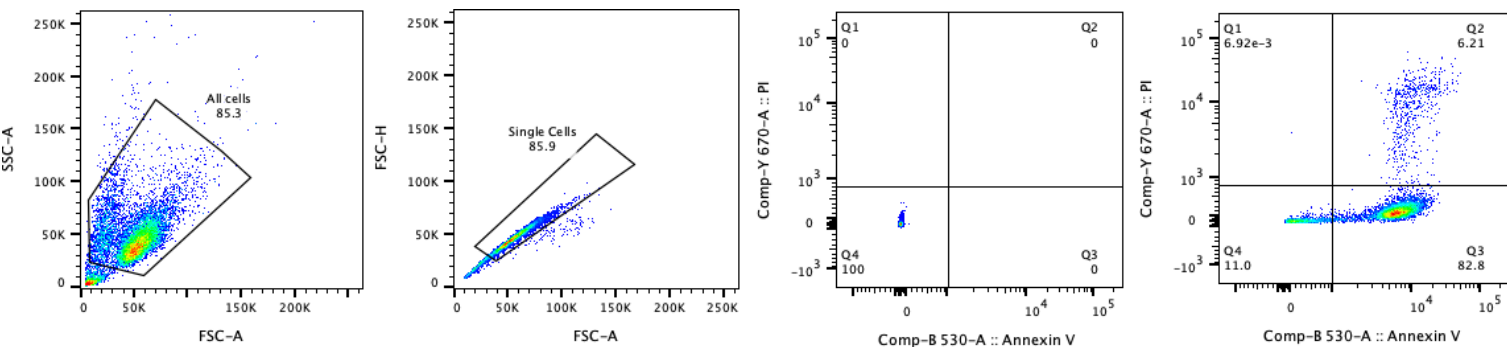**b**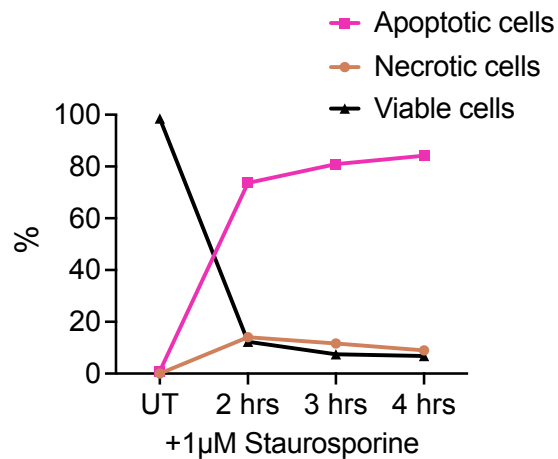**c**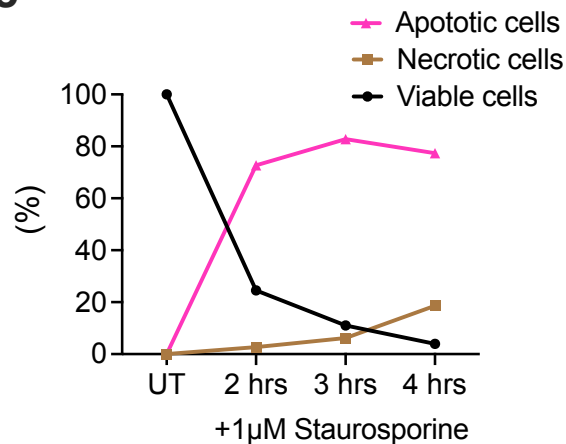

Supplement: Supplementary file 1 — Figure S1: Generation of apoptotic cells. Thymocytes from wild type mice or human lymphocyte Jurkat cells were treated with 1 μM staurosporine for 2, 3, or 4 h to induce apoptosis. (a) Flowcytometry gating strategy; (b) Percentage of apoptotic (Red), Necrotic (brown) and viable (black line) thymocytes and (c) apoptotic Jurkat cells are shown. [file FSB2-40-e72156-s001.pdf]

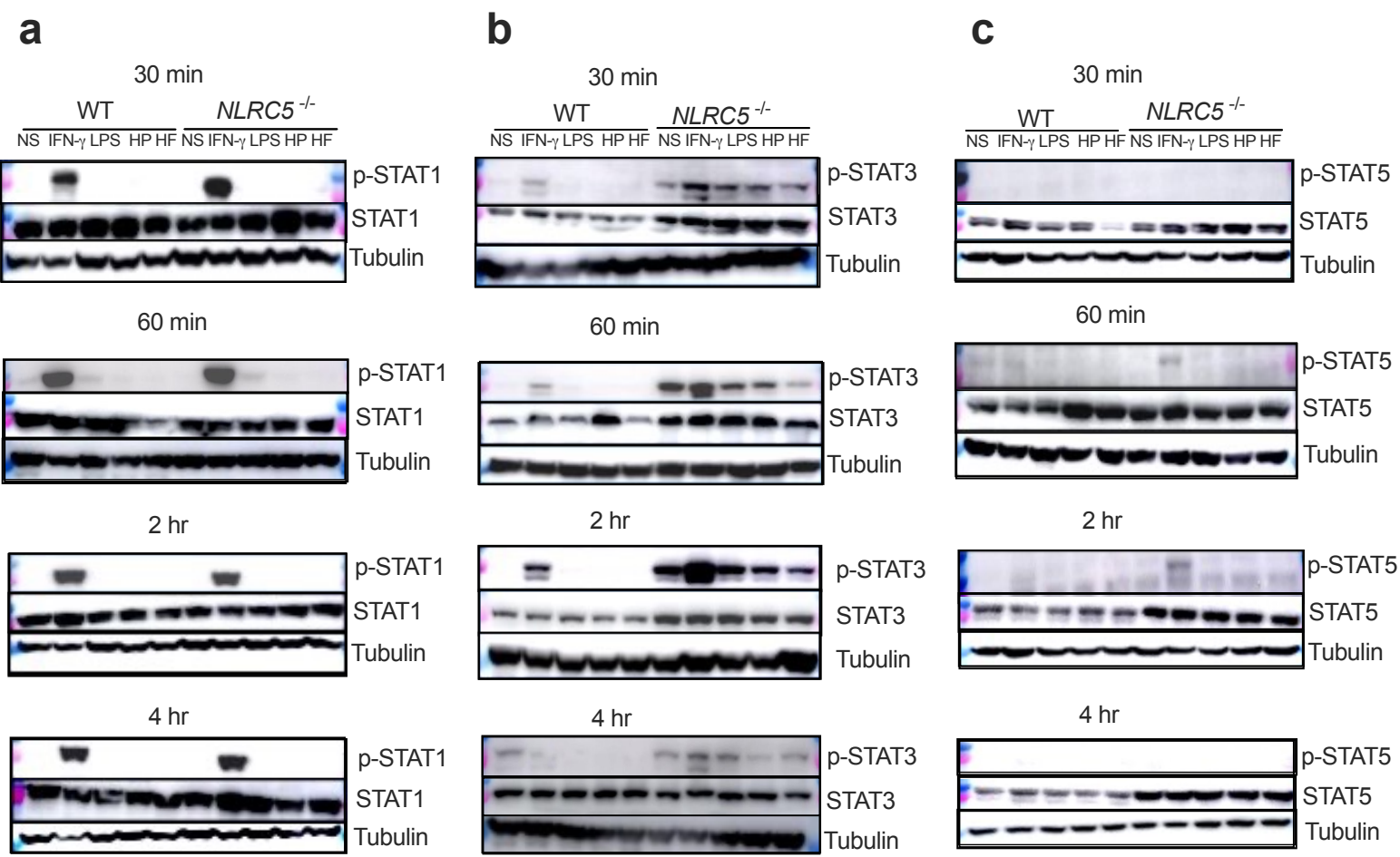

Supp Fig. 3

Supplement: Supplementary file 2 — Figure S2: Pro‐inflammatory cytokine responses are not significantly increased in Nlrc5 −/− macrophages. Expression of (a) Ifnb, (b) Il1b, (c) Il6, and (d) Tnf in mouse splenic macrophages from WT and Nlrc5 mø‐KO mice. Gene expression was normalized to that of Gapdh. Cells were either not‐stimulated (NS) or stimulated with either LPS (100 ng/mL), H. pylori (HP), H. felis (HF) (both MOI = 10) for 2 h. Data are presented as the means ± SEM for triplicate determinations from n = 3–4 biological replicates and analyzed by two‐way ANOVA. [file FSB2-40-e72156-s007.pdf]

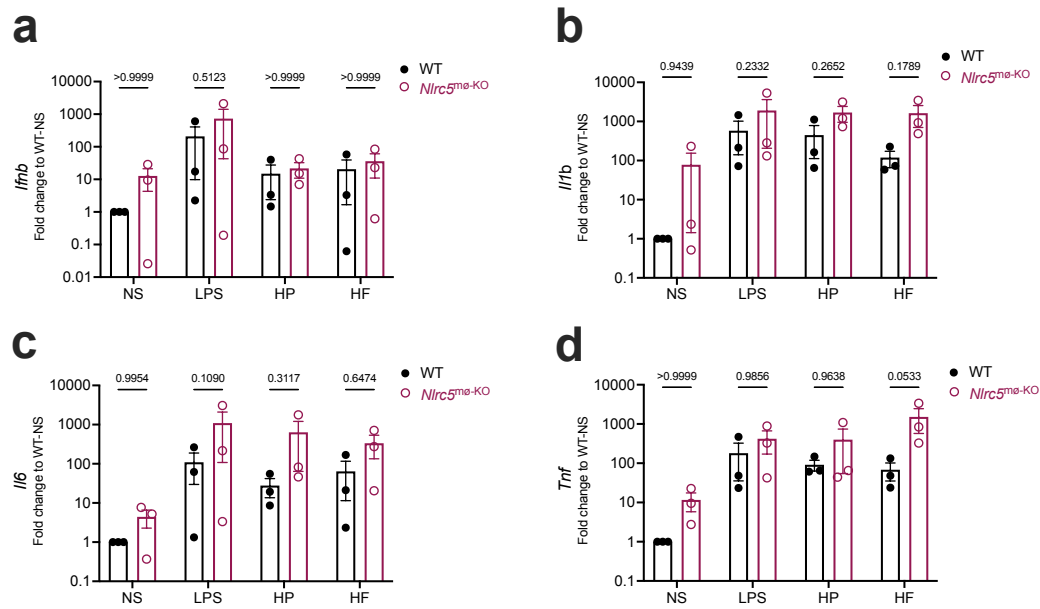

Supp Fig. 2

Supplement: Supplementary file 3 — Figure S3: Increased phosphorylation of STAT3 but not STAT1 or STAT5 in macrophages lacking functional NLRC5. Total and phosphorylated forms of (a) STAT1(Tyr701), (b) STAT3(Tyr705), and (c) p‐STAT5(Tyr694) were detected by Western blotting in WT and NLRC5 −/− THP‐1 macrophages. Cells were either not‐stimulated (NS) or stimulated with IFN‐γ (100 ng/mL), LPS (100 ng/mL), H. pylori (HP) or H. felis (HF) (both MOI = 10) for 30 or 60 min, or for 2 or 4 h. Representative Western blot images are shown for n = 3 independent experiments. [file FSB2-40-e72156-s006.pdf]

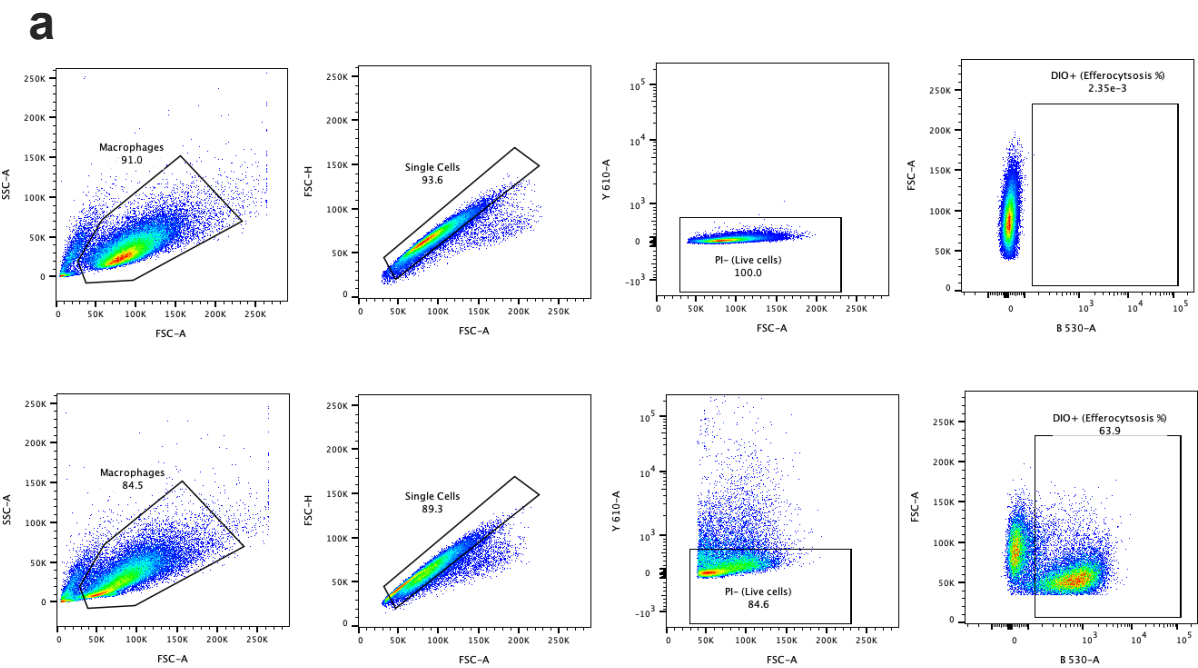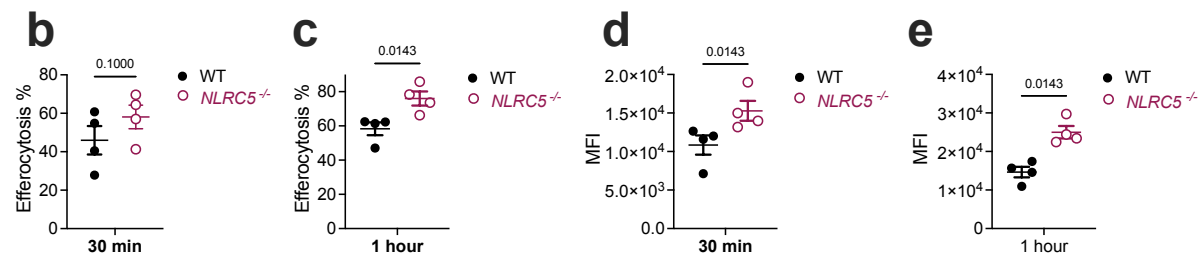

Supp Fig. 4

Supplement: Supplementary file 4 — Figure S4: Increased efferocytosis of apoptotic T cells in macrophages lacking functional NLRC5. WT or NLRC5 −/− THP‐1 macrophages were cocultured with DiO‐labeled apoptotic Jurkat cells (1:1 ratio). (a) Flow cytometry gating strategy. Efferocytosis was measured by flow cytometry and expressed as (b, c) the percentage of DiO+ cells (efferocytosis %) or (d, e) MFI at 30 min or 1 h. Data are presented as the means ± SEM for triplicate determinations from n = 4 biological replicates and analyzed by the Mann–Whitney U test. [file FSB2-40-e72156-s004.pdf]

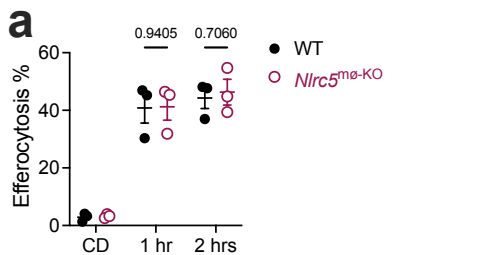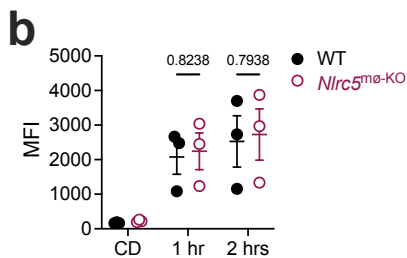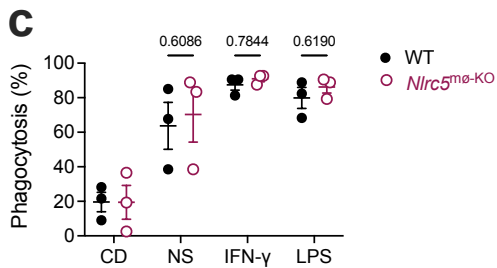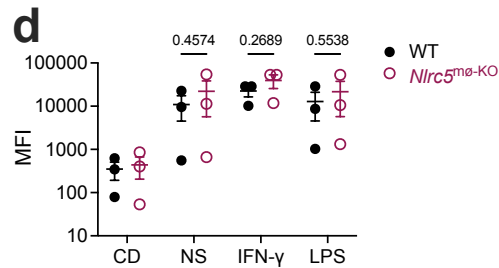

Supp Fig.5

Supplement: Supplementary file 5 — Figure S5: Functional Nlrc5 deficiency does not result in increased efferocytosis or phagocytosis in mouse BMDMs. Bacterial phagocytosis and efferocytosis was measured by flow cytometry. (a, b) BMDMs from WT or Nlrc5 mø‐KO mice were cocultured with DiO‐labeled apoptotic mouse thymocytes (1:1 ratio) and expressed as efferocytosis % and MFI at 1‐ or 2‐h. (c, d) BMDMs from WT or Nlrc5 mø‐KO mice were cocultured with DiO‐labeled H. pylori (MOI = 10) and expressed as bacterial phagocytosis % and MFI in either not‐stimulated (NS) or stimulated with either IFN‐γ or LPS (both 100 ng/mL). Data are shown as the mean ± SEM and the presented values are combined from 3 independent experiments. Two‐way ANOVA. CD, Cytochalasin D. [file FSB2-40-e72156-s008.pdf]

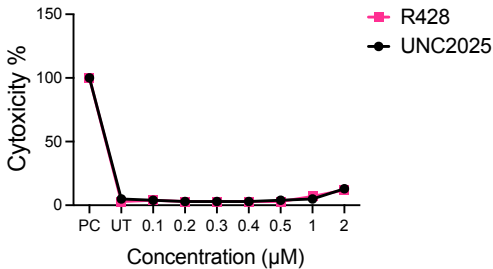

Supp Fig.6

Supplement: Supplementary file 6 — Figure S6: Cytotoxicity level of the small molecules R428 and UNC2025. THP‐1 macrophages were left untreated (UT) or treated with either R428 or UNC2025 for 2 h at the indicated concentrations. Lactate dehydrogenase (LDH) released into the culture media was determined using a Cytotoxicity Detection Kit (Promega, G1780). Cytotoxicity level of R428 (Red line) and UNC2025 (Black line). PC, positive control. [file FSB2-40-e72156-s005.pdf]

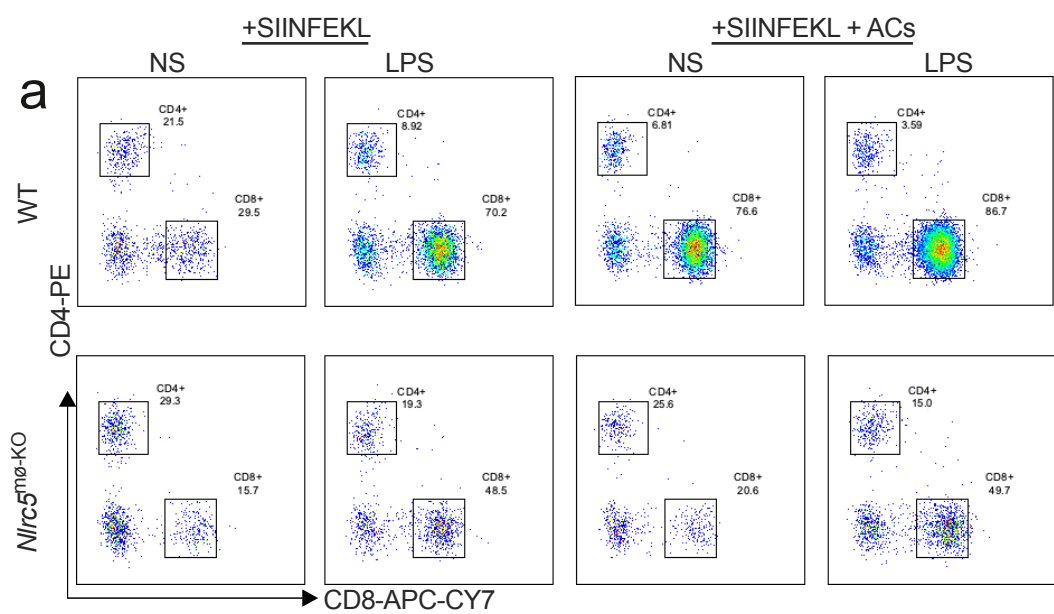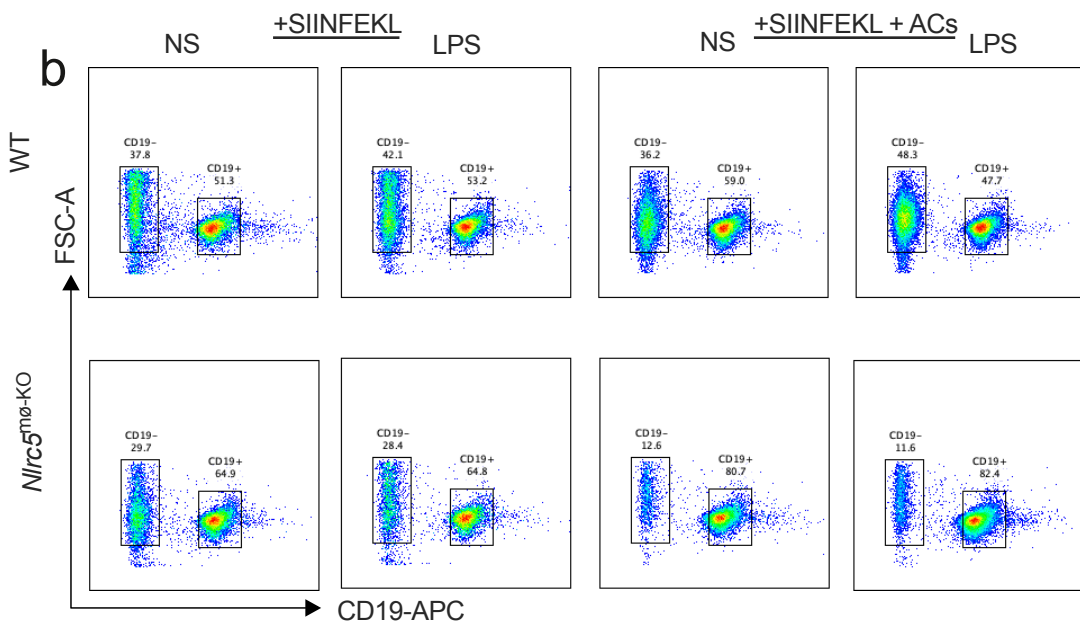

Supp Fig. 7

Supplement: Supplementary file 7 — Figure S7: MHC‐I‐restricted antigen presentation is reduced in macrophages lacking functional Nlrc5. Macrophages that had been either left unstimulated (NS) or pre‐stimulated with LPS (100 ng/mL) were cocultured with either soluble SIINFEKL or SIINFEKL‐loaded apoptotic mouse thymocytes, then co‐incubated with CFSE labeled OT‐I cells for 72 h. (a) Gating of CD4+ T cells and CD8+ T cells. (b) Percentages of CD8+ T cells that had undergone cell division, as observed by a reduction in CFSE staining. (c) Percentages of CD19+ B cells that had been cocultured with splenic macrophages from WT or Nlrc5 mø‐KO mice. Representative images from three independent experiments. [file FSB2-40-e72156-s002.pdf]
