# Supplementary material for: NLRC5‐Deficient Macrophages Promote a Tumor‐Permissive Phenotype via AXL‐ and MERTK‐Mediated Efferocytosis
Source: FASEB J. 2026 Aug 1;40(15):e72156. doi: 10.1096/fj.202504879R (PMC13428295; doi:10.1096/fj.202504879R)
Supplement: Supplementary file 8 — Table S1: Antibody information for Western blotting. Table S2: Primer sequences used for qPCR analysis (Human). Table S3: Primer sequences used for qPCR analysis (Mouse). Table S4: Antibody information for flowcytometry analyses. [file FSB2-40-e72156-s003.docx]

**Supplementary materials**

**Supplemental Table 1. Antibody information for Western blotting**

| Antibody | Reactivity | Isotype | Dilution | Catalogue | Manufacturer |
| --- | --- | --- | --- | --- | --- |
| STAT1 | H M R | Rabbit | 1:1000 | 9172T | CST |
| P-STAT1^(Tyr701)^ | H M R | Rabbit | 1:1000 | 9171 | CST |
| STAT3 (D1B2J) | H M R | Rabbit | 1:1000 | 30835T | CST |
| P-STAT3^(Tyr705)^ (D3A7) | H M R | Rabbit | 1:2000 | 9145T | CST |
| STAT5 (D2O6Y) | H M R | Rabbit | 1:1000 | 94205S | CST |
| P-STAT5^(Tyr694)^ (C11C5) | H M R | Rabbit | 1:1000 | 9359S | CST |
| MERTK Mab (1C11) | H | Rabbit | 1:1000 | BSM-52273R | ThermoFisher |
| MERTK^(Tyr749/753/754)^ | H M R | Rabbit | 1:1000 | P186-749T | PhosphoSolutions |
| Anti-AXL | H M R | Rabbit | 1:1000 | PA5-106118 | ThermoFisher |
| P-AXL ^(Tyr702)^ | H M R | Rabbit | 1:1000 | PA5-64862 | ThermoFisher |
| Anti-Tubulin (YL1/2) | H M R | Rat | 1:2000 | ab6160 | Abcam |
| Goat anti-Rabbit IgG Secondary Antibody, HRP | R | Goat | 1:2000 | 31460 | ThermoFisher |
| Goat anti-Rat IgG Secondary Antibody, HRP | Rat | Goat | 1:2000 | A18865 | ThermoFisher |

**H**= human; **M**= mouse; **R**= rabbit

**Supplemental Table 2. Primer sequences used for qPCR analysis (Human)**

| No- | Gene | Forward sequence | Reverse sequence |
| --- | --- | --- | --- |
| 1 | *GAPDH* | ACCACCCTGTTGCTGTAGCCAA | GTCTCCTCTGACTTCAACAGCG |
| 2 | *AXL* | AGGCAGGCAGTGCCAAATCC | CATAGCCAGGCTGGGACACG |
| 3 | *MERTK* | GACCGTGTCCAAGGGAGTGC | CCTGCTGCTTAGTCGGAGGC |
| 4 | *TYRO3* | GACTGGTCCTGAGAGGGTGA | ATGGCACACCTTCTACCGTG |

**Supplemental Table 3. Primer sequences used for qPCR analysis (Mouse)**

| No- | Gene | Forward sequence | Reverse sequence |
| --- | --- | --- | --- |
| 1 | *Gapdh* | GAACGGGAAGCTTGTCATCAA | CTAAGCAGTTGGTGGTGCAG |
| 2 | *Axl* | CCAACACCTGAGCGGCAAGA | TGGGCCTTTGCATTTGTGGC |
| 3 | *Mertk* | TTGGCGGCCTCAGCAAGAAA | GAGCGCTGCACACTGGCTAT |
| 4 | *Tyro3* | GGACTGGCTTCTCTGCTGCTC | CTCTGTGCGCTGGGTCACTC |
| 5 | *Socs1* | GAGTAGGATGGTAGCACGCAA | TAATCGGAGTGGGAGCGGAA |
| 6 | *Socs3* | GGACCAAGAACCTACGCATCCA | CACCAGCTTGAGTACACAGTCG |
| 7 | *Ifnb* | CCCTATGGAGATGACGGAGA | ACCCAGTGCTGGAGAAATTG |
| 7 | *Il1b* | ACGGACCCCAAAAGATGAAG | TTCTCCACAGCCACAATGAG |
| 8 | *Il6* | ATGGATGCTACCAAACTGGAT | TGAAGGACTCTGGCTTTGTCT |
| 9 | *Tnf* | CAAATTCGAGTGACAAGCCTG | GAGATCCATGCCGTTGGC |
| 10 | *Il10* | CGGGAAGACAATAACTGCACCC | CGGTTAGCAGTATGTTGTCCAGC |
| 11 | *Tgfb* | TGATACGCCTGAGTGGCTGTCT | CACAAGAGCAGTGAGCGCTGAA |

**Supplemental Table 4. Antibody information for flow cytometry analyses.**

|  | Flurophores | Dilution | Catalogue | Company |
| --- | --- | --- | --- | --- |
| CD19 | APC | 1:100 | 152409 | Biolegend |
| CD4 | PE | 1:100 | 553652 | BD Bioscience |
| CD8a | APC-Cy7 | 1:100 | 557654 | BD Bioscience |
| PI |  | 1:2000 | 421301 | Biolegend |

**Supplementary Figure 1: Generation of apoptotic cells.** Thymocytes from wild type mice or human lymphocyte Jurkat cells were treated with 1 µM staurosporine for 2, 3 or 4 hours to induce apoptosis. **(a)** Flowcytometry gating strategy; **(b)** Percentage of apoptotic (Red), Necrotic (brown) and viable (black line) thymocytes and **(c)** apoptotic Jurkat cells are shown.

**Supplementary Figure 2. Increased phosphorylation of STAT3 but not STAT1 or STAT5 in macrophages lacking functional NLRC5.** Total and phosphorylated forms of **(a)** STAT1^(Tyr701)^, **(b)** STAT3^(Tyr705)^ and **(c)** p-STAT5^(Tyr694)^ were detected by Western blotting in WT and *NLRC5^–/–^*  THP-1 macrophages. Cells were either not-stimulated (NS) or stimulated with IFN-γ (100 ng/ml), LPS (100 ng/ml), *H. pylori* (HP) or *H. felis* (HF) (both MOI=10) for 30 or 60 minutes, or for 2 or 4 hours. Representative Western blot images are shown for n=3 independent experiments.

**Supplementary Figure 3**: **Pro-inflammatory cytokine responses are not significantly increased in *Nlrc5*^–/–^ macrophages.** Expression of **(a)** *Ifnb,* **(b)** *Il1b*, **(c)** *Il6* and **(d)** *Tnf* in mouse splenic macrophages from WT and *Nlrc5*^mø-KO^ mice. Gene expression was normalized to that of *Gapdh.* Cells were either not-stimulated (NS) or stimulated with either LPS (100 ng/ml), *H. pylori* (HP), *H. felis* (HF) (both MOI=10) for 2 hours. Data are presented as the means ± SEM for triplicate determinations from n=3-4 biological replicates and analysed by two-way ANOVA.

**Supplementary Figure 4. Increased efferocytosis of apoptotic T cells in macrophages lacking functional NLRC5.** WT or *NLRC5*^–/–^ THP-1 macrophages were co-cultured with DiO-labelled apoptotic Jurkat cells (1:1 ratio). **(a)** Flow cytometry gating strategy. Efferocytosis was measured by flow cytometry and expressed as **(b, c)** the percentage of DiO^+^ cells (efferocytosis %) or **(d, e)** MFI at 30 min or 1 hours. Data are presented as the means ± SEM for triplicate determinations from n=4 biological replicates and analysed by the Mann-Whitney U test.

**Supplementary Figure 5: Functional Nlrc5 deficiency does not result in increased** **efferocytosis or phagocytosis in mouse BMDMs.** Bacterial phagocytosis and efferocytosis was measured by flow cytometry. **(a, b)** BMDMs from WT or *Nlrc5*^mø-KO^ mice were co-cultured with DiO-labelled apoptotic mouse thymocytes (1:1 ratio) and expressed as efferocytosis % and MFI at 1- or 2-hours. **(c, d)** BMDMs from WT or *Nlrc5*^mø-KO^ mice were co-cultured with DiO-labelled *H. pylori* (MOI=10) and expressed as bacterial phagocytosis % and MFI in either not-stimulated (NS) or stimulated with either IFN-γ or LPS (both 100 ng/ml). Data are shown as the mean ± SEM and the presented values are combined from 3 independent experiments. Two-way ANOVA. CD= Cytochalasin D.

**Supplementary Figure 6: Cytotoxicity level of the small molecules R428 and UNC2025.** THP-1 macrophages were left untreated (UT) or treated with either R428 or UNC2025 for 2 hours at the indicated concentrations. Lactate dehydrogenase (LDH) released into the culture media was determined using a Cytotoxicity Detection Kit (Promega, G1780). Cytotoxicity level of R428 (Red line) and UNC2025 (Black line). PC=positive control

**Supplementary Figure 7. MHC-I-restricted antigen presentation is reduced in macrophages lacking functional Nlrc5.** Macrophages that had been either left unstimulated (NS) or pre-stimulated with LPS (100 ng/ml) were co-cultured with either soluble SIINFEKL or SIINFEKL-loaded apoptotic mouse thymocytes, then co-incubated with CFSE labelled OT-I cells for 72 hours. **(a)** Gating of CD4^+^ T cells and CD8^+^ T cells. **(b)** Percentages of CD8^+^ T cells that had undergone cell division, as observed by a reduction in CFSE staining. **(c)** Percentages of CD19^+^ B cells that had been co-cultured with splenic macrophages from WT or *Nlrc5*^mø-KO^ mice. Representative images from 3 independent experiments
